# Medical Education and the London University

**Published:** 1907-11-30

**Authors:** 


### The Hospital

A Journal of

The Medical Sciences and Hospital Administration.

New Series. No. 36, Vol. II. [No. 1108, Vol. XLIII.] SATURDAY, NOVEMBER 30, 1907.

### MEDICAL EDUCATION AND THE LONDON UNIVERSITY.

At the meeting of the Senate of the University of London held on Wednesday, November 20, a report was received from the committee specially appointed to advise the Senate on the course to be pursued in regard to the proposed Institute of Medical Sciences at South Kensington. The committee came to the following conclusions:—(*a*) That, owing to the lack of adequate financial support, the scheme for the establishment of an Institute of Medical Sciences, as set forth in the original appeal and (in a modified form) in the appeal of June 1905, has proved abortive; (*b*) that, apart from the money difficulty, which in the opinion of the committee is of itself fatal, the scheme has also become impracticable for other reasons. The Medical Faculty, which formerly reported in favour of the scheme, has now reported against it. Several of the medical schools have changed their opinions in the same sense, and some of them have made arrangements involving considerable outlay for providing more efficient instruction in preliminary and intermediate medical studies; (*c*) that, in the above circumstances, the University has no claim to the money which has already been paid by subscribers, or to the fulfilment of promises by subscribers who have not yet paid their subscriptions; and (*d*) that, in the absence of any special directions in any particular case, all subscriptions already paid ought at once to be returned to the donors (including in that term the executors or legal representatives of deceased donors) without any suggestion as to any possible application of the money to any other purpose.

The Senate consequently resolved to communicate with the donors to the Medical Institute Fund in accordance with these conclusions, informing them that the money paid would be placed at their disposal.

Thus is buried the failure of a fine project. It has been pointed out previously in the columns of The Hospital that the original scheme in the minds of its creators was that of a single institute in a central part of the Metropolis, accessible to every medical school and favouring none, it being a necessary corollary to the erection of such a centre that as long as University and King's Colleges were directly or indirectly connected with any individual hospital they also should abandon the teaching of the preliminary and intermediate medical studies. It is needless here to repeat how the University abandoned this fine ideal; how University College was incorporated without really severing its intimate connection with the hospital on the other side of Gower Street; how the site proposed was geographically most unsuitable; how the proposed institute dwindled from one requiring half a million of money to one which should be built for seventy thousand pounds; and, finally, how it has become apparent that the larger and more efficient medical schools were asked to assist in the erection of what was practically another small medical school which would compete with them unfairly, owing to the advantage of financial support from the funds of the University and the privilege of internal examinations; all these events have been responsible for a change of opinion in the three largest London schools from cordial support to determined hostility.

The Senate, having been well advised by an influential lay committee, has taken the only possible course open to it, and is to be congratulated on the wisdom of its decision. In order to obtain any measure of success for such a revolution it was a necessity that either the proposals adopted should be of immediate or ultimate advantage to the schools affected or that, should it be necessary that they should suffer for the general good, they should all suffer to the same extent. As it was apparent that this was not the case, success was out of the question and the scheme was abortive.

The election of a member to represent the Faculty of Medicine upon the Senate in the place of Dr. Lauriston Shaw took place the following day, and was interesting from the fact that the question upon which the election was to have been fought—namely, the proposed erection of the third centre at South Kensington—was settled upon the eve of the election, and when the conflicting parties arrived upon the following day, there was nothing left to fight about. In no previous contest has there been such a copious supply of election literature, but the very free discussion of the whole subject was of undoubted benefit. By far the most important result of much assertion and counter-assertion was a letter from the Principal of the University, Sir Arthur Rücker, making it quite clear that the University had never pledged itself, either actually or morally, to build an institute at

South Kensington as part of a bargain by which St. George's Hospital Medical School had abandoned the teaching of the preliminary and intermediate medical studies. This impression had been created at a previous meeting of the Faculty of Medicine by a speech made by the Dean of St. George's Hospital Medical School and by subsequent correspondence from the Treasurer of the Hospital and two previous Deans.

The candidates for election were three: Professor Ernest Starling, of University College, who was in favour of the proposed institute; Mr. F. C. Wallis, of Charing Cross Hospital, who was opposed both to its erection and to the election of another member of the staff of University College or Hospital upon the Senate, as they were already over-represented with six members; and Dr. Norman Moore, of St. Bartholomew's Hospital, who was repeating a candidature which had been unsuccessful at the previous Senatorial election, his policy being practically the same as that of Mr. Wallis. Needless to say, the division of his opponents' vote gave the election to Professor Starling, though on the first ballot he would have been defeated by a narrow majority had not the opposing vote been split.

Though Dr. Starling's election to the Senate is not so unfortunate as it might have been had not the question of concentration been previously settled, the excessive representation of University College is to be deplored as not making for the equitable consideration of many outstanding problems, not the least of which is the privileged position of University College itself with regard to certain preliminary medical examinations.

